# Features of Atherosclerosis in the Tunica Adventitia of Coronary and Carotid Arteries in a Black Kenyan Population

**DOI:** 10.1155/2014/456741

**Published:** 2014-03-17

**Authors:** Julius Ogeng'o, Kevin Ongeti, Moses Obimbo, Beda Olabu, Philip Mwachaka

**Affiliations:** Department of Human Anatomy, University of Nairobi, P.O. Box 30197, Nairobi 00100, Kenya

## Abstract

*Introduction*. Histologic changes which occur in the tunica adventitia during initiation, progression, and complications of atherosclerosis are seldom reported. This study aimed at describing the features of atherosclerosis in the tunica adventitia of two of the commonly afflicted arteries, namely, left anterior descending coronary and common carotid in black Kenyans. *Materials and Methods*. Specimens from 108 individuals [76 males and 32 females, mean age 34.6] were processed for paraffin embedding. Seven micron thick sections were stained with Mason's trichrome and Haematoxylin/Eosin and examined with a light microscope. *Results*. Features of atherosclerosis were present in the tunica adventitia of 14.8% of left anterior descending arteries and 11.1% of common carotid arteries. Increase in adventitial thickness was associated with increased density of vasa vasora in 8.3% of both arteries. In the left anterior descending and common carotid arteries, 6.5% and 3.7% of cases, respectively, the tunica adventitia thickened without intimal hyperplasia. *Conclusion*. Features of atherosclerosis occur in the tunica adventitia of coronary and carotid arteries in over 10% of the black Kenyans studied. These features often precede the intimo medial changes. Tunica adventitia should therefore be prioritized in evaluation for atherosclerosis, in individuals at risk. This may enhance early detection and intervention.

## 1. Introduction

Tunica adventitia of arteries was previously believed to be involved only in physical and nutritive support of the vessel wall [1]. Currently, however, it is known to be a highly cellular and metabolically active component of the vessel wall, capable of controlling its structure, function, and disease processes from “outside-in” [2–4]. It plays a leading role in the initiation, progression, and complications of atherosclerosis [5, 6]. Adventitial thickness has been independently correlated with cardiovascular risk factor profile [7–9]. Consequently, it has become a target for therapeutic interventions in the treatment of atherosclerosis [10, 11]. In spite of these developments, there are few histological reports of the changes which occur in the tunica adventitia of vulnerable arteries. Further, the adventitia role in atherosclerosis and the stage of involvement are not universally accepted, as some studies suggest it is not involved [12]. As atherosclerosis increases in Sub-Saharan Africa, there is need to document these adventitial features so as to improve understanding of the disease process as well as aid in early diagnosis and intervention. This study therefore examined changes in tunica adventitia of both left anterior descending (LAD) and common carotid arteries (CCA), in a black Kenyan population.

## 2. Materials and Methods

Materials for this study were obtained from proximal left anterior descending arteries and distal common carotid arteries of 108 black Kenyans [76 males, 32 females; of mean age 34.6 years; range 2–82 years], during autopsy. Samples were taken within 48 hours of death, to avoid overt postmortem damage to the tissues. Two millimeter long specimens were fixed by immersion in 10% formaldehyde solution for three days, then trimmed, and processed routinely for paraffin embedding. Seven micrometer sections were cut and stained with Haematoxylin/Eosin for demonstration of the general organization of the mural components and Mason's trichrome stain for demonstration of collagen and smooth muscle cells. The slides were then examined at various magnifications, using a light microscope and images taken using a high resolution digital camera. Results are presented in micrographs.

## 3. Results

**Figure 1:**
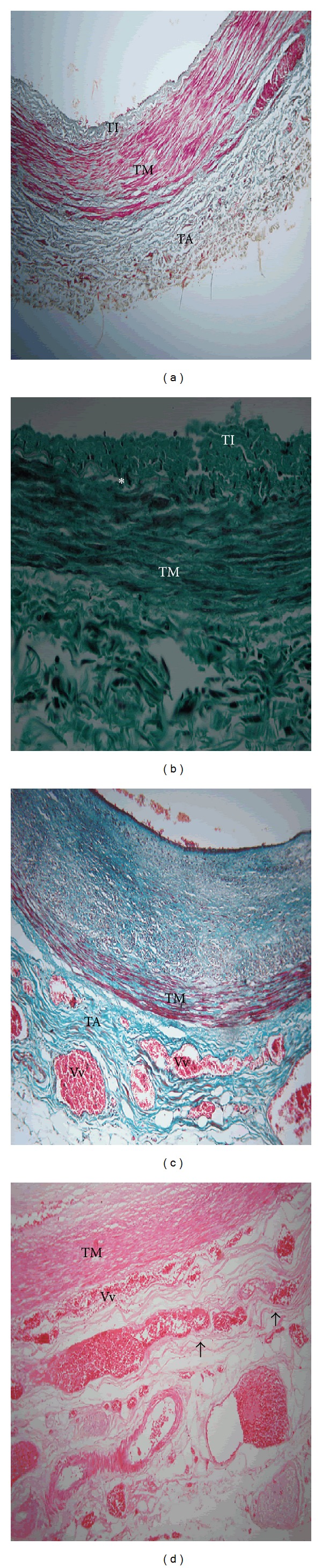
Full wall thickness of proximal LAD in black Kenyans showing features of atherosclerosis in the tunica adventitia. TA: tunica adventitia, TM: tunica media, and TI: tunica intima. (a) Thickening of tunica adventitia in a 35-year-old male. Note that the TA is thick when tunica intima is only slightly thickened. Mason's trichrome ×40. (b) Thickened tunica adventitia in a 62-year-old female associated with mild intimal hyperplasia and disintegration of internal elastic lamina (asterisk). Mason's trichrome ×100. (c) Increased density of vasa vasora (Vv) in thickened tunica adventitia. Note that it is associated with severe intimal hyperplasia and increased prominence of perivascular adipose tissue. Mason's trichrome ×100. (d) Increased vasa vasora (Vv) density in tunica adventitia. Note the vasa vasora penetrating into the tunica media and splitting some of the outer layers of smooth muscle (arrows). Haematoxylin/Eosin ×100.

Tunica adventitia was thickened in 16 (14.8%) and 12 (11.1%) of the LAD and CCA, respectively. In 7 (6.5%) cases of LAD, adventitial thickening was not associated with increased vasa vasora density and occurred with normal tunica intima (Figure 1(a)). In 8 (7.4%) cases, the thickening was associated with mild intimal hyperplasia and disintegration of internal elastic lamina (Figure 1(b)). In 9 (8.3%) cases, the tunica adventitial thickening was associated with marked increase in density of vasa vasora and intimal hyperplasia (Figure 1(c)). In 3 (2.8%) cases, the vasa vasora penetrated into the tunica media, splitting the outer layers of smooth muscle cells (Figure 1(d)).

**Figure 2:**
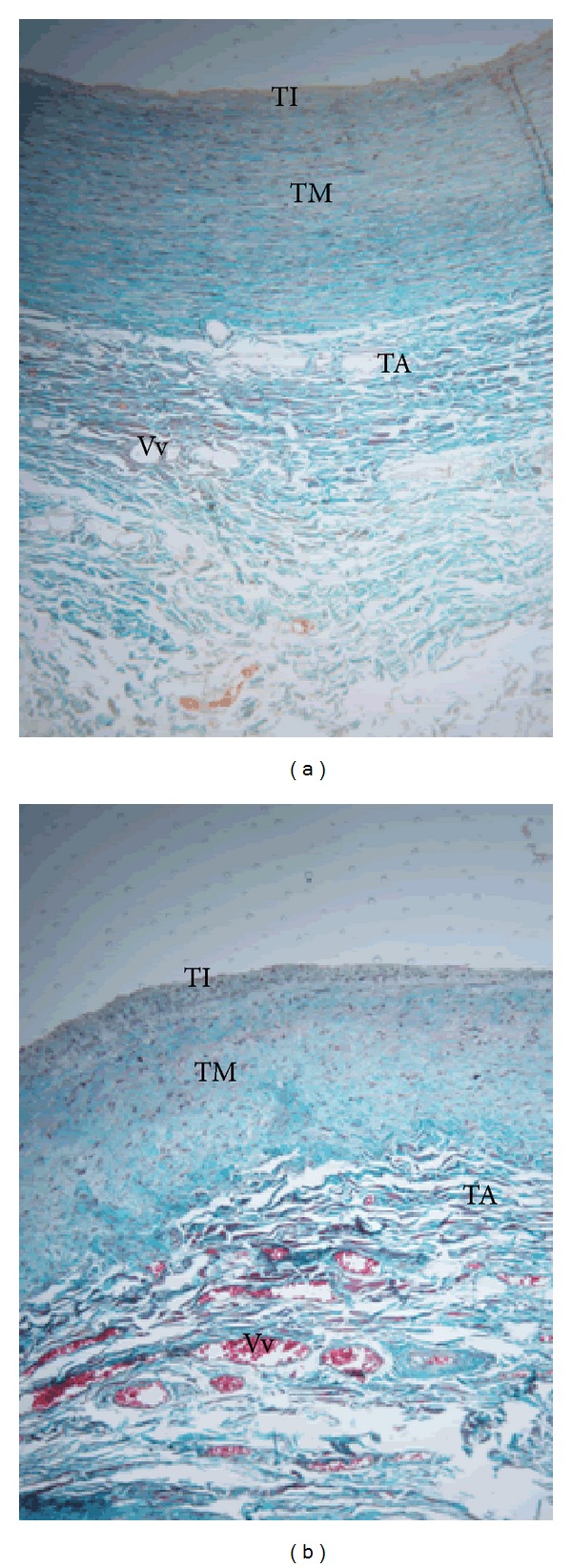
Full wall thickness of common carotid artery in black Kenyans showing features of atherosclerosis in the tunica adventitia. TA: tunica adventitia, TM: tunica media, and TI: tunica intima. (a) Thickened tunica adventitia (TA) with increased density of vasa vasora (Vv) in 57-year-old male. Note a thin tunica intima (TI). Mason's trichrome ×100. (b) Thickened tunica adventitia (TA), with increased density of vasa vasora (Vv) associated with medial degeneration in a 47-year-old female. Note the unstructured nature of tunica media (TM). Mason's trichrome ×100.

In the CCA, similar features were observed. Only 3 (2.8%) cases of tunica adventitial thickening were associated with intimal thickening. In 8 (8.3%) of the cases, thickening of the tunica adventitia was associated with only slight intimal thickening. The thickening was associated with increased density of vasa vasora in 6 (5.5%) cases. Additionally, however, in 4 (3.7%) cases, marked thickening of the adventitia with proliferation of vasa vasora occurred without intimal hyperplasia (Figure 2(a)). Further, in 3 (2.8%) cases, proliferation of vasa vasora in the tunica adventitia was associated with medial degeneration whereby the tunica media appeared unstructured (Figure 2(b)).

## 4. Discussion

Observations of the current study reveal that marked thickening of tunica adventitia occurred in over 10% of the cases. Adventitial thickening is known to increase during atherosclerosis [13, 14]. This increase in the thickness is thought to be due to activation of adventitial fibroblasts by atherogenic stimuli, leading to production of more extracellular matrix. The activated fibroblasts also upregulate production of chemokines and cytokines that lead to recruitment of inflammatory cells [2, 15]. The findings of the present study imply that a significant proportion of asymptomatic individuals in the Kenyan population display features of atherosclerosis. Accordingly, screening for atherosclerosis in individuals at risk of the disease should also include the tunica adventitia.

A remarkable finding of the present study was that, in a substantial proportion, increased thickness of tunica adventitia occurred in the absence of intimal hyperplasia, which is well known to herald atherosclerosis [16, 17]. This appears at variance with the generally accepted view that adventitial thickening occurs in advanced atherosclerosis because of inflammation and increased neovascularization by vasa vasora [17, 18]. This early isolated adventitial thickening is consistent with the outside-in mechanism of atherogenesis in which the inflammation is initiated in the adventitia as the first responder early in the disease process and progresses inwards towards the intima [3, 19, 20]. Accordingly, in evaluating vulnerable arteries, due attention should be paid to both the tunica adventitia and intima for features of early atherosclerosis.

Adventitial vasa vasora proliferation, usually triggered by atherogenic stimuli such as hypertension, dyslipidemia, and hypoxia, occurs in the pathogenesis, distribution, progression, and development of complications of atherosclerosis [11, 21, 22]. Their density increases with growth of atherosclerotic plaque in asymptomatic patients and is correlated with plaque vulnerability and haemorrhage [11, 23, 24]. The findings of the current study that, in 8.3% of LAD and 5.5% of CCA, increased tunica adventitial thickening occurred in the wake of high vasa vasora density suggest that, in this proportion of asymptomatic patients, atherosclerosis was present and in some cases had attained complicated stages and cardiovascular events were imminent.

Proliferation of adventitial vasa vasora and their invasion into the tunica media and intima, called mural neovascularization, as observed in the present study, facilitate pathogenesis of atherosclerosis by providing a considerable endothelial exchange surface for harmful circulating substances and cells to the vessel wall [25–27]. Further, they create a conduit for transport of inflammatory cells and mediators into the arterial wall to promote chronic inflammation and plaque neovascularization [21, 28, 29]. The increased density of vasa vasora within the thickened tunica adventitia observed in the present study suggests that, in patients at risk of atherosclerosis, there is need for early evaluation of tunica adventitial integrity and commencement of appropriate measures to avert complications.

## 5. Conclusion

Findings of the present study reveal that features of atherosclerosis, namely, increased adventitial thickness and vasa vasora density, occur in the tunica adventitia of coronary and carotid arteries in over 10% of the black Kenyans studied. These features often precede intimal and medial changes. Tunica adventitia should therefore be prioritized in evaluation of vulnerable arteries for atherosclerosis, in individuals at risk. This may enhance early detection and intervention.
